# Local stiffness and work function variations of hexagonal boron nitride on Cu(111)

**DOI:** 10.3762/bjnano.12.46

**Published:** 2021-06-17

**Authors:** Abhishek Grewal, Yuqi Wang, Matthias Münks, Klaus Kern, Markus Ternes

**Affiliations:** 1 Max Planck Institut für Festkörperforschung, Heisenbergstrasse 1, D-70569 Stuttgart, Germany; 2 Peter Grünberg Institute, Forschungszentrum Jülich, D-52425 Jülich, Germany; 3 Institut de Physique, École Polytechnique Fédérale de Lausanne, CH-1015 Lausanne, Switzerland; 4 II. Institute of Physics, RWTH Aachen University, D-52074 Aachen, Germany

**Keywords:** decoupling layers, hexagonal boron nitride, local stiffness, Moiré superstructure, work function variation

## Abstract

Combined scanning tunnelling and atomic force microscopy using a qPlus sensor enables the measurement of electronic and mechanic properties of two-dimensional materials at the nanoscale. In this work, we study hexagonal boron nitride (*h*-BN), an atomically thin 2D layer, that is van der Waals-coupled to a Cu(111) surface. The system is of interest as a decoupling layer for functional 2D heterostructures due to the preservation of the *h*-BN bandgap and as a template for atomic and molecular adsorbates owing to its local electronic trapping potential due to the in-plane electric field. We obtain work function (Φ) variations on the *h*-BN/Cu(111) superstructure of the order of 100 meV using two independent methods, namely the shift of field emission resonances and the contact potential difference measured by Kelvin probe force microscopy. Using 3D force profiles of the same area we determine the relative stiffness of the Moiré region allowing us to analyse both electronic and mechanical properties of the 2D layer simultaneously. We obtain a sheet stiffness of 9.4 ± 0.9 N·m^−1^, which is one order of magnitude higher than the one obtained for *h*-BN/Rh(111). Using constant force maps we are able to derive height profiles of *h*-BN/Cu(111) showing that the system has a corrugation of 0.6 ± 0.2 Å, which helps to demystify the discussion around the flatness of the *h*-BN/Cu(111) substrate.

## Introduction

Two-dimensional hexagonal boron nitride (*h*-BN) is among the list of materials that garnered tremendous interest following the exfoliation of mono- and few-layer thick graphene films [1–2]. Unique properties, such as high thermal stability and conductivity, immense intra-sheet stiffness, and excellent dielectric properties, make *h*-BN interesting for technological applications. For example, thin films of *h*-BN have been used as a passivating layer for graphene and MoS_2_-based electronics utilising the small lattice mismatch, the large optical phonon modes, and particularly the large bandgap [3–10]. Furthermore, when grown on metal substrates *h*-BN can be used as a nanoscale template for atoms, molecules, and nanostructures with well-controlled adsorption and electronic properties [11–18]. In such systems, *h*-BN shows a rich structural and electronic morphology, which depends on the lattice mismatch and the interaction strength with the substrate: Large and flat lattice-matched terraces for *h*-BN/Ni(111) [19–20], strain-induced highly corrugated layers for *h*-BN/Rh(111) [21–23], and template layers for molecules with strong local variations of the work function for *h*-BN/Ir(111) [24] are representative of such morphological diversity.

We use low-temperature combined scanning tunnelling (STM) and non-contact atomic force microscopy (nc-AFM) to study *h*-BN on Cu(111). This template has interesting properties because the dielectric layer is only very weakly bound to the metal and shows an electronically induced Moiré superstructure [25–26]. First STM studies on this system pointed to only a small geometrical corrugation [27]. Further experimental investigations, using both local probes and averaging techniques, revealed more details of the mechanical and electronic properties of the system, but also inconsistent results about the structural corrugation [26,28–30]. For example, Brülke et al. used high-resolution low-energy electron diffraction and normal incidence X-ray standing wave techniques to detect the large separation of 3.24 Å between the *h*-BN sheet and the topmost Cu(111) layer [29]. They found almost no height difference between B and N atoms and excluded significant buckling perpendicular to the surface. Interestingly, this is in contrast to measurements by Schwarz et al. who used a more local analysis of the corrugation by exploiting nc-AFM concluding an absolute height difference of 0.3–0.7 Å between “rim” and “valley” sites of the spatially corrugated monolayer [26]. Recently, however, Zhang et al. used STM in combination with DFT simulations to study the variation of the local work function and bandgap within the Moiré superlattice and found that the variation depends on the angle of the Moiré with respect to the substrate lattice, but inferred only marginal structure modulation [30].

To shed more light on this controversy we use an alternative method to verify the mechanical properties of the monolayer by measuring the stiffness of the *h*-BN layer at different locations of the superstructure and comparing these results with concomitantly recorded local work function variations. We determine the stiffness of the system by mapping and comparing the short-range interaction forces between the monolayer and the probing metallic tip [31]. This technique enables us to detect the sheet stiffness with unprecedented spatial resolution [23]. On *h*-BN/Rh(111), a different system than the one studied in this work, the extremely low stiffness of only approx. 1 N·m^−1^ at the weakly bound rim areas confirmed the buckling of the monolayer into the third dimension to relieve the strain induced by the significant lattice mismatch of this strongly corrugated van der Waals layer [23].

## Results and Discussion

### STM/AFM on *h*-BN/Cu(111)

**Figure 1:**
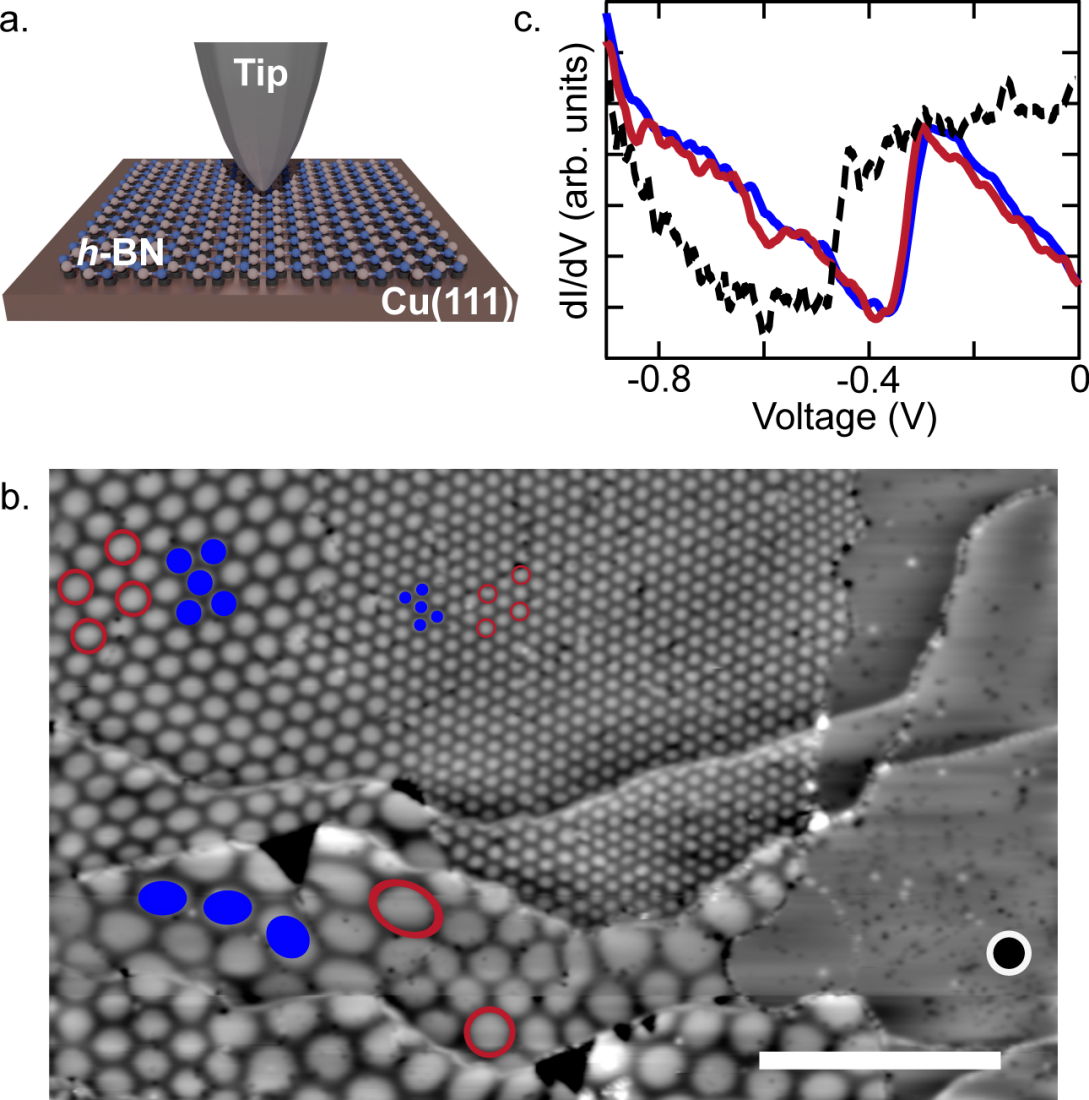
(a.) Scheme of the experiment. (b.) Large-scale (200 × 125 nm^2^) constant-current (*I* = 20 pA, *V* = 3.7 V) STM topography of *h*-BN/Cu(111) and the bare Cu(111) surface. Blue circles and red rings mark exemplary valley and rim areas, respectively. (c.) Differential conductance d*I*/d*V* spectra taken at rim (red line) and valley (blue line) sites and at the bare Cu(111) substrate (dashed black line).

As illustrated in Figure 1a, we employ nc-AFM to probe the electronic and topographic structure of a monolayer of *h*-BN on a Cu(111) surface. Figure 1b shows a typical large-scale constant-current STM scan of this structure. We observe the monolayer growing over step edges of the underlying Cu(111) substrate. Weak interlayer interaction allows the van der Waals layer to have varying relative rotational orientations, θ = 0–4°, on the substrate corresponding to a Moiré pattern wavelength of λ = 3–14 nm. Furthermore, we observe an upward shift of the surface state onset of Cu(111) from approx. −455 meV on the bare substrate to approx. −320 meV on *h*-BN/Cu(111) (Figure 1c) [32]. We found this shift to vary only marginally (approx. ±10 meV) with the Moiré periodicity or between rim and valley sites [33–34].

**Figure 2:**
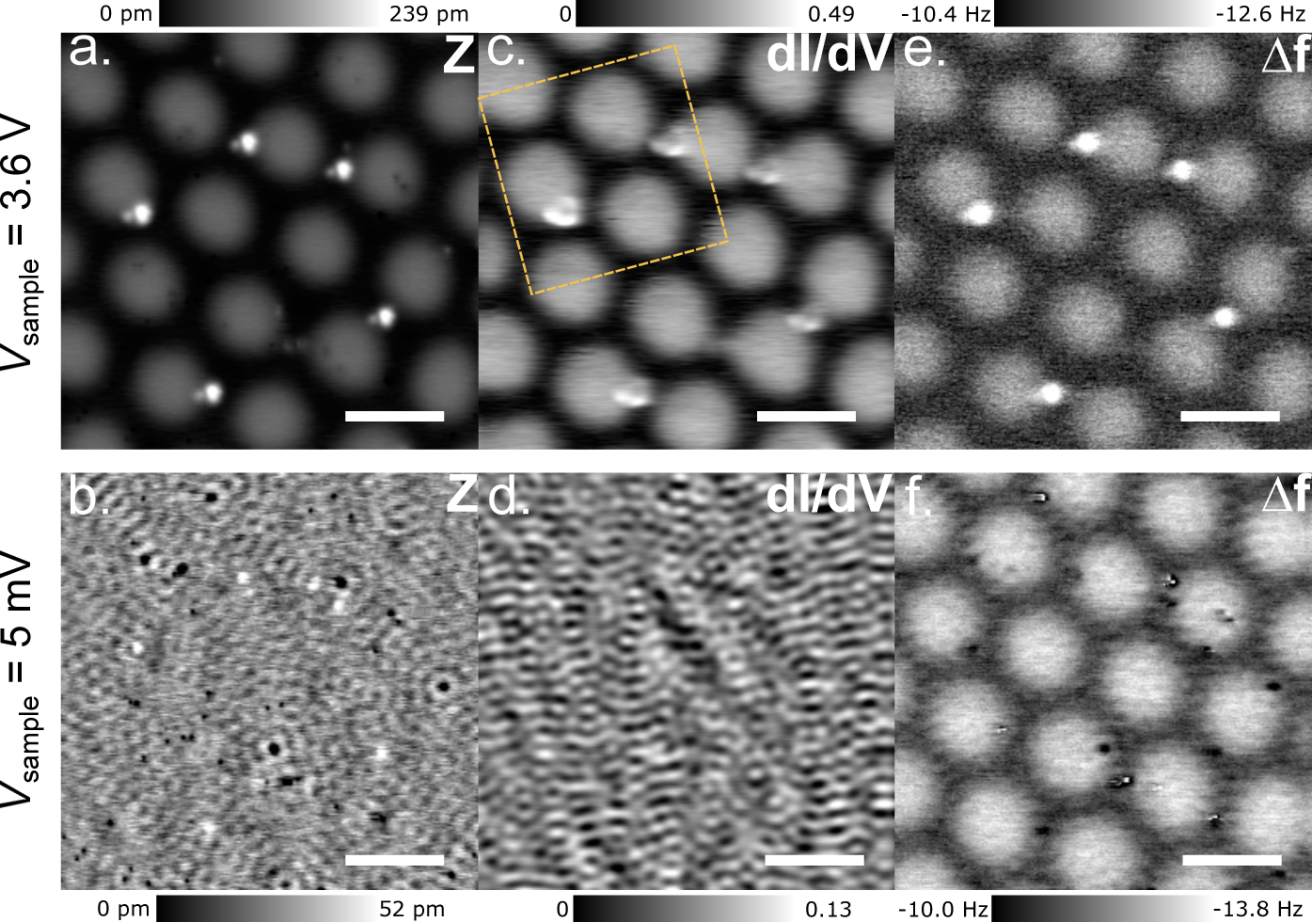
STM/AFM characterisation of a *h*-BN/Cu(111) Moiré superstructure. (a., b.) Constant-current topography at *I* = 500 pA and *V* = 3.6 V (top) or *V* = 5 mV (bottom). (c., d.) Simultaneously measured differential conductance (d*I*/d*V*) maps (*V*_mod_ = 10 mV (top) and *V*_mod_ = 1 mV (bottom). (e., f.) Frequency shift (Δ*f*) maps (*A*_osc_ = 50 pm). The dashed yellow box marks the area used for the Φ maps in Figure 3 (see below). Scale bar: 10 nm.

*h*-BN/Cu(111) is known to have an indirect bandgap of 6.1 eV [35], which can be modulated by the Moiré pattern [30]. We analyse the substrate using STM topography, dI/d*V*, and frequency shift, Δ*f*, AFM maps under low (in-gap) and high (conduction band onset) bias conditions (see Figure 2). Due to *h*-BN being insulating, no spectroscopic contribution is expected at low bias voltages making it transparent to STM, as seen in Figure 2b,d. At this bias, only Friedel oscillations due to the scattering of the Cu(111) surface-state electrons on defects and adsorbates are observed. Contrarily, as Figure 2a reveals, at higher bias, the STM topography corresponds to the modulation of the *h*-BN/Cu(111) interface state as we will show below.

Despite the large change in electronic density of states and, thus, tip height between the data obtained at the two different sample biases, we observe a one-to-one correspondence between the simultaneously recorded Δ*f* images and the STM topography. Also, the Δ*f* variation between rim and valley areas in both images changes only marginally. The additionally imaged adsorbates (dots or ring-like features) allow, thereby, the precise alignment between the subsequently acquired data sets.

### Work function variation

While the work function is generally discussed in the framework of a macroscopic quantity [36], we will use the notation, valid also on the nanoscale, that Φ is the local surface potential measured from the Fermi level, *E*_F_ [37]. For a nanoscale patterned surface, such as *h*-BN/Cu(111), Φ fluctuations can originate from a locally varying charge transfer between the substrate and the dielectric layer [38–40].

In our studied substrate, it is the lattice mismatch between *h*-BN and the Cu(111) substrate that leads to a varying atomic registry and subsequently induces a lateral modulation of the charge transfer [41]. Additionally, this leads to in-plane electric fields, which have been shown to trap atoms, molecules, and nanoclusters [11,13,42].

**Figure 3:**
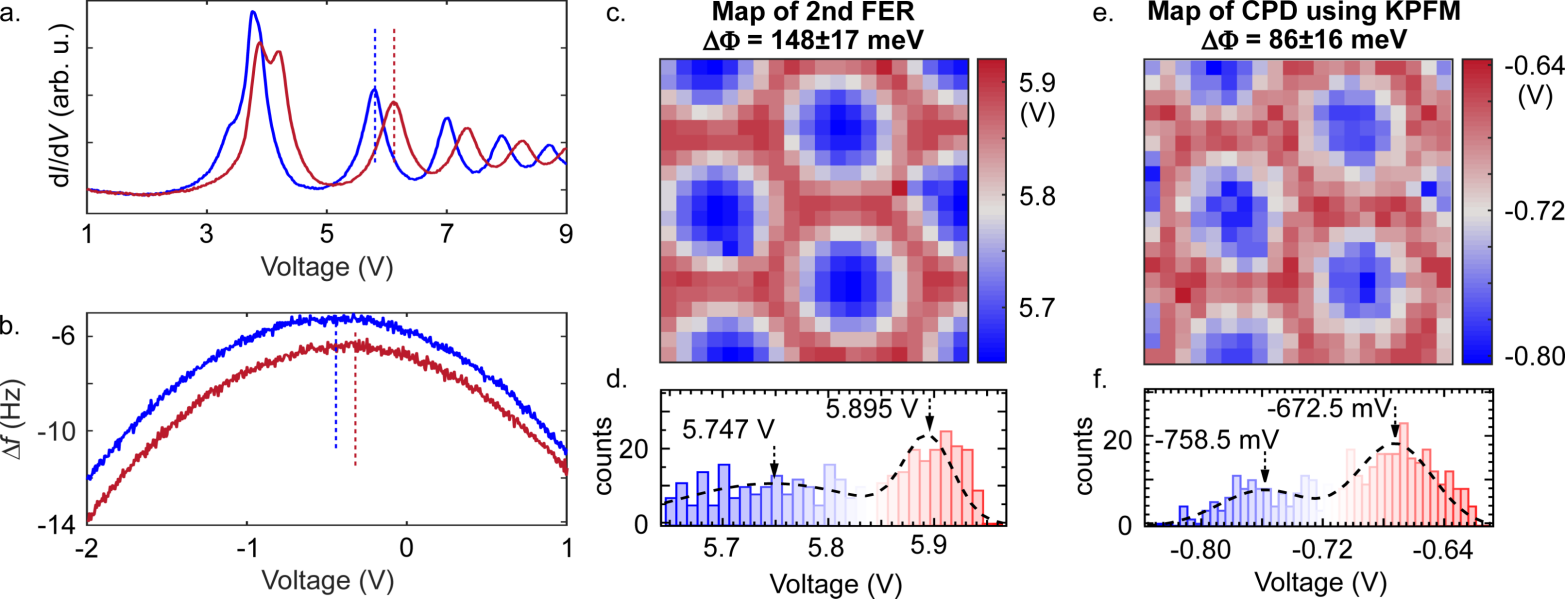
Work function variation between rim (red) and valley (blue) areas measured using (a.) d*I*/d*V* at constant current (*I* = 100 pA) and (b.) KPFM at constant height (stabilised in the valley at *I* = 100 pA, *V* = 10 mV, *A*_osc_ = 50 pm), respectively. The dotted vertical lines mark exemplary FER and CPD values used for the spatially resolved plots shown in (c.) and (e.). The maps are recorded at the yellow box indicated in Figure 2c on a 20 × 20 grid over 20 × 20 nm^2^. They display the position of the maximum of the second peak in the FER (c.) and the maximum of the KPFM parabola (e.), respectively. (d., f.) Histograms and fits for rim and valley where arrows mark the centre positions of the Gaussians used for the determination of the distribution centre.

To map the local Φ fluctuations and to correlate them with the structural properties of the surface, we use two complementary methods: The first method is based on a shift of the field emission resonance (FER) induced by Φ variations. The effective potential well of depth Φ at the surface of a metal can accommodate a series of Rydberg states, extending a few angstroms into the vacuum above the metal surface [43]. These image potential states (IPSs), states arising from the long-range image potential experienced by an electron in front of a metal surface, are delocalised in the surface plane and contain the full band of the 2D electron gas. However, the electric field exerted by the proximity of the probing tip distorts the energy spacing of the IPSs. These distorted IPSs are referred as FER, which are revealed in dI/d*V* measurements as strong peaks at positive bias. [43]. Figure 3a shows such spectra in which we observe a series of peaks whose energies are strongly influenced by the measurement position. The non-trivial double peak structure at 3.5–4.5 V is due to varying contributions from the two interfaces of the dielectric layer. We therefore evaluate the unambiguous shift of the second peak at around 5.6–6.0 V as a measure for the local Φ variation.

Our nc-AFM allows us to employ with Kelvin probe force microscopy (KPFM) a second, independent method to detect the variation in Φ. For this we record the frequency shift, Δ*f*, of the resonance frequency of the cantilever oscillating perpendicular to the surface as a function of the bias voltage (see Figure 3b). At the extrema of the parabolic Δ*f* curves, the electrostatic force is minimised by the applied voltage, which compensates the contact potential difference between Φ of the tip and Φ of the sample [44].

Using the shift of the FER we find an average variation between valley and rim regions of ΔΦ = 148 ± 17 meV, which agrees well with previous observations [27,45]. Interestingly, however, we find a significantly smaller average difference between valley and rim regions of only ΔΦ = 86 ± 16 meV when analysing the contact potential difference (CPD) data. This hints toward a lower lateral resolution of the KPFM measurement compared to the FER map. The Δ*f* signal in KPFM originates from the relatively long-ranged electrostatic interaction, which is therefore a weighted average over the relevant size of the tip (radii of 5–10 nm [46]). It is of the same order as the size of the rim and valley regions and, as a result, leads to an underestimation of ΔΦ. Nevertheless, both measurement techniques agree well in their qualitative results as it is evident from the ΔΦ maps (see Figure 3c,e).

### Stiffness

Probing the force perpendicular to the substrate, *F*_⟂_, at varying tip–sample separations *z*, the effective stiffness of a nanostructure can be evaluated by comparing the *F*_⟂_(*z*) behaviour at different areas of the Moiré superstructure [23]. Additionally, such a set of data enables us to obtain maps of constant tip–sample interaction forces that allow for the quantification of the corrugation of the Moiré superstructure.

**Figure 4:**
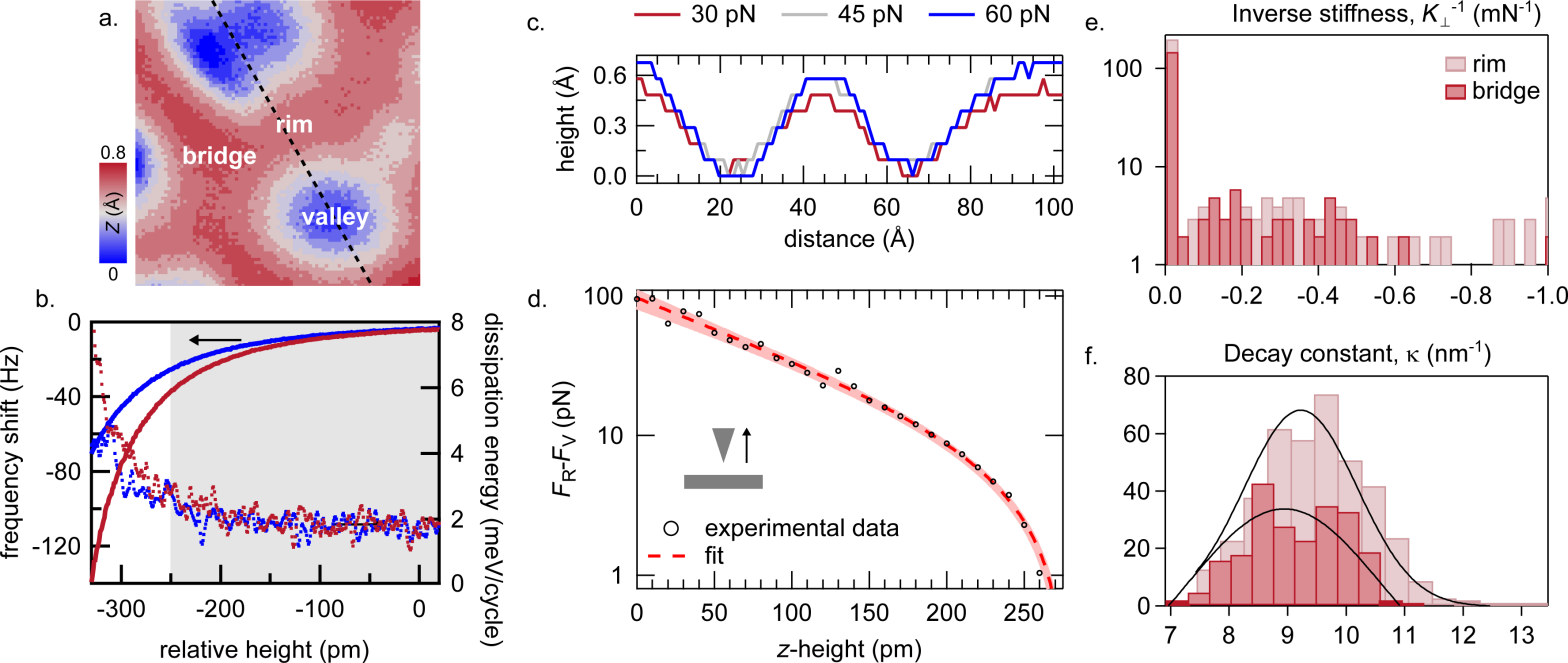
Local stiffness of *h*-BN/Cu(111). (a.) Topography of an 8 × 8 nm^2^ *h*-BN/Cu(111) area corresponding to a constant force *F*_⟂_ = 30 pN. (b.) Point Δ*f* and excitation energy vs relative *z*-height curve obtained at rim (red) and valley (blue) positions. The grey shaded area marks the *z*-range used for the Δ*f* maps. (c.) Line profiles taken from constant-vertical-force maps along the black dashed line in (a.), at *F*_⟂_ = 30 pN (red), 45 pN (grey), and 60 pN (blue), respectively. (d.) Average attractive short-range force obtained for the rim and the bridge region after subtracting the contribution from valley area (experimental data) and fit. The red area marks the 90% confidence range. (e., f.) Histograms of inverse stiffness (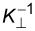) and decay constant (κ) of the rim (pink) and bridge sites (red), respectively.

To obtain such data we map the Δ*f* signal at constant oscillation amplitude for an 8 × 8 nm^2^ area at 28 relative tip–surface distances. From the set point, *I* = 100 pA, *V* = 10 mV, we approach the tip by 250 pm (defined as *z* = 0 pm), set *V* = 0 mV, and record Δ*f* maps as the tip is retracted from *z* = 0 to 270 pm, with a 10 pm spacing (grey shaded area in Figure 4b).

Using the matrix inversion method [47], we convert the 3D stack of Δ*f* data into the out-of-surface force component *F*_⟂_. The now obtained 3D force stack enables us to evaluate the interaction between the tip and the monolayer substrate without being strongly influenced by the electronic corrugation as in STM-only measurements. By taking a 2D cut at constant force through the 3D stack, we obtain a topography at a constant tip–substrate interaction force, which allows us to visualise the corrugation between rim and valley areas (see Figure 4a). Figure 4c shows different line profiles corresponding to constant force values of *F*_⟂_ = 30, 45, and 60 pN. These line profiles reveal an average corrugation of 0.6 ± 0.2 Å, which agrees well with the corrugation of 0.3–0.7 Å obtained by Schwarz and co-workers [26]. In these line profiles we obtain a minimal corrugation increase at increased constant force values, which hints to some mechanical relaxations of the rim areas under the influence of the force exerted by the tip.

To analyse this effect we separate the short-range forces, which act between the tip apex and the sample and which vary over the corrugation of the monolayer, from electrostatic and van der Waals long-range forces by subtracting the average total force *F*_V_ measured in the valley areas (blue regions in Figure 4a) from the total forces *F*_R_ acting at rim and bridge sites of the superstructure (red regions in Figure 4a), assuming infinite stiffness for valley areas. An example of Δ*f*-vs-*z* point spectra obtained at valley and rim regions is shown in Figure 4b. The grey shaded area, showing little change in dissipation, is used for the *z*-range for which Δ*f* maps are acquired. The resulting difference *F*_D_ = *F*_R_ − *F*_V_ is the additional short-range force, which solely influences rim and bridge areas and which might locally lift the *h*-BN layer leading to an increase of corrugation. Figure 4d shows *F*_D_(*z*) averaged over rim and bridge sites, which decays with *z* mainly exponentially as expected from an interatomic short-range force when neglecting Pauli repulsion [23,48]. The over-exponential decay at *z >* 200 pm is caused by a small offset of *F*_0_ ≈ 1 pN due to the finite set of Δ*f* data, which results in *F*_R_ ≡ *F*_V_ at the last measurement height (*z* = 270 pm, see Experimental section). A very soft *h*-BN layer would show an additional over-exponential increase at small *z* due to the lifting of the sheet by 
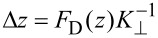
, where *K*_⟂_ is the local vertical stiffness [23]. Assuming an exponential decay of the intrinsic short-range force between tip an substrate and compensating for any lifting, we get for the local vertical force, *F*_D_, as a function of relative height:

[1] $$F_{\text{D}}\left(z\right)=\left(\kappa /K_{⊥}\right)\times W_{0}\left[F_{0}\kappa /K_{⊥}\exp\left(-\kappa z\right)\right]+F_{0},$$

where *W*_0_ is the real-valued branch of the Lambert *W* function and κ is the decay constant [23]. As shown in Figure 4d, we obtain a good agreement between our data and the model. The best fit yields an average local vertical stiffness of *K*_⟂_ = 9.4 ± 0.9 N·m^−1^ (Figure 4d). This demonstrates the high stiffness (negligible softness) of the *h*-BN monolayer on Cu(111), which is one order of magnitude higher than that found on Rh(111) [23]. The statistical evaluation of the spatial variation of *K*_⟂_ is shown in Figure 4e. The dramatic peak at small inverse stiffness in both rim and bridge areas means an almost perfect exponential behaviour of the short-range force and that *h*-BN/Cu(111) undergoes no significant deformation. Also, the histogram of the decay constant κ in Figure 4f reveals only negligible differences between rim (κ = 9.2 ± 1.3 nm^−1^) and bridge areas (κ = 8.9 ± 1.4 nm^−1^) indicating almost no difference in the mechanical properties between different areas of the Moiré superstructure. Additionally, the cluster of 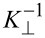 values close to 0 m·N^−1^ suggests that the short range forces between rim and bridge sites are almost identical.

## Conclusion

We report the electronic and mechanical characterisation of *h*-BN/Cu(111) using an STM/AFM. Our STM studies corroborate that the *h*-BN monolayer is only weakly coupled to the Cu(111) surface as is evidenced by the large angular range of Moiré superstructures observed, which in turn leads to work function patterning. Using FER and KPFM maps we report a work function variation of 148 ± 17 and 86 ± 16 meV, respectively, which agrees well with the previous experimental and theoretical studies [27,45].

3D force maps, obtained via constant-height Δ*f* imaging, allow us to test the mechanical stability of the monolayer substrate in the short-range force regime. Using the AFM tip as a nanoindenter we probe its effect on the *h*-BN/Cu(111) system. We obtain a sheet stiffness of *K*_⟂_ = 9.4 ± 0.9 N·m^−1^, which is one order of magnitude larger than that obtained on *h*-BN/Rh(111), indicating substantial mechanical stability. The small lattice mismatch between *h*-BN and Cu(111), compared to *h*-BN and Rh(111), results in lower strain and no buckling of the substrate and, thus, to high stiffness. Furthermore, our results corroborate that *h*-BN/Cu(111) has a small corrugation of 0.6 ± 0.2 Å but is mechanically stiff making it an appealing platform for studying intrinsic electronic and mechanical properties of nanostructures.

## Experimental

We employ a custom-built ultrahigh-vacuum (below 10^−10^ mbar) low-temperature (*T* = 1.4 K) nc-AFM operated in frequency-modulated mode. A stiff qPlus cantilever design [49] (*k*_0_ = 1800 N·m^−1^, *f*_0_ = 29077 Hz, *Q* = 60000) at an oscillation amplitude *A*_osc_ = 50 pm enables the nc-AFM functionality. We calibrated the amplitude prior to the measurement atop the bare Cu(111) substrate [50]. The bias voltage *V* is applied to the substrate and the tunnelling current *I* is measured at the virtually grounded tip. The STM/AFM images were processed with the Gwyddion software [51].

**FER and KPFM measurements:** FER measurements are taken by modulating *V* (*f*_m_ = 607 Hz, *V*_m_ = 10 mV peak-to-peak) and detecting the d*I*/d*V* signal with the lock-in technique while the tip height is adjusted so that the current *I* remains constant (constant-current mode) during the bias sweep. For KPFM measurements we stabilise the tip height at *I* = 100 pA and *V* = 10 mV. We then record the frequency shift Δ*f* with respect to *f*_0_ while *V* is swept at constant tip height.

**Vertical stiffness:** The 3D Δ*f* data (8 × 8 × 0.27 nm^3^), evaluated in this work, are obtained by taking 28 2D maps at successively increased tip–sample separation (Δ*z* = 10 pm) starting from a tip height stabilised at *I* = 100 pA, *V* = 10 mV in the valley area and approaching the tip by −250 pm at *V* = 0 mV. We define this *z*-height as *z* = 0. We use the known exponential dependence of the average current on the tip retraction to compensate for any z-drift over the approx. 23 h of data acquisition.

**Sample preparation:** A Cu(111) single crystal (MaTeck GmbH) is cleaned via repeated cycles of Ar-ion sputtering at room temperature followed by annealing to 1020 K in an ultrahigh-vacuum preparation chamber. A partial layer of *h*-BN is grown by chemical vapour deposition by heating the Cu(111) sample to 980 K and exposing it to 25 L of borazine (HBNH_3_) gas (Katchem spol s.r.o.). *h*-BN grows in a self-terminating growth process [19]. It is then transferred in situ to the nc-AFM for characterisation.
